# RETRACTED: Zafar et al. *Moringa concanensis*-Mediated Synthesis and Characterizations of Ciprofloxacin Encapsulated into Ag/TiO_2_/Fe_2_O_3_/CS Nanocomposite: A Therapeutic Solution against Multidrug Resistant *E. coli* Strains of Livestock Infectious Diseases. *Pharmaceutics* 2022, *14*, 1719

**DOI:** 10.3390/pharmaceutics17111481

**Published:** 2025-11-17

**Authors:** Naheed Zafar, Bushra Uzair, Farid Menaa, Barkat Ali Khan, Muhammad Bilal Khan Niazi, Fatima S. Alaryani, Kamlah Ali Majrashi, Shamaila Sajjad

**Affiliations:** 1 Department of Biological Sciences, International Islamic University Islamabad, Islamabad 44000, Pakistan; 2 Department of Internal Medicine and Nanomedicine, California Innovations Corporation, San Diego, CA 92037, USA; 3 Department of Pharmacy, Gomal University, Dera Ismail Khan 29050, Pakistan; 4 School of Chemical and Materials Engineering, National University of Sciences and Technology (NUST), Islamabad 44000, Pakistan; 5 Department of Biology, College of Science, University of Jeddah, Jeddah 21589, Saudi Arabia; 6 Biological Sciences Department, College of Science & Arts, King Abdulaziz University, Rabigh 21911, Saudi Arabia; 7 Department of Physics, International Islamic University, Islamabad 44000, Pakistan

The Journal retracts the article “*Moringa concanensis*-Mediated Synthesis and Characterizations of Ciprofloxacin Encapsulated into Ag/TiO_2_/Fe_2_O_3_/CS Nanocomposite: A Therapeutic Solution against Multidrug Resistant *E. coli* Strains of Livestock Infectious Diseases.” [1], cited above.

Following publication, concerns were brought to the attention of the Editorial Office regarding the integrity of Figure 12 in this publication [1].

Adhering to our standard procedure, an investigation was conducted by the Editorial Office and Editorial Board that identified a duplication between sub-figures 12c and 12b presented in this article [1] and earlier publications [2,3] produced by a different research group and presenting different experimental conditions. While the authors collaborated within this process, raw material meeting the journal’s requirements for original images (https://www.mdpi.com/journal/molecules/instructions#oriimages) could not be provided for Editorial Board evaluation. Consequently, the Editorial Board has lost confidence in the reliability of the findings and has decided to retract this publication, as per MDPI’s retraction policy (https://www.mdpi.com/ethics#_bookmark30).

This retraction was approved by the Editor-in-Chief of the journal *Pharmaceutics*.

The authors did not agree to this retraction.
